# Open Reduction and Internal Fixation of Dorsal Fracture-Dislocation of the Distal Interphalangeal Joint Using a Low-Profile Mini-Plate: A Report of Three Cases

**DOI:** 10.7759/cureus.77260

**Published:** 2025-01-10

**Authors:** Masayoshi Ikeda, Yuka Kobayashi, Daisuke Nakajima, Ikuo Saito, Takayuki Ishii

**Affiliations:** 1 Department of Orthopaedic Surgery, Shonan Central Hospital, Fujisawa, JPN; 2 Department of Orthopaedic Surgery, Tokai University Hachioji Hospital, Hachioji, JPN; 3 Department of Orthopaedic Surgery, Isehara Kyodo Hospital, Isehara, JPN; 4 Department of Orthopaedic Surgery, Seirei Fuji Hospital, Shizuoka, JPN

**Keywords:** distal interphalangeal joint, distal phalangeal base fracture, dorsal fracture dislocation, internal fixation, plate fixation, surgical technique

## Abstract

Although dorsal fracture-dislocations of the distal interphalangeal (DIP) joints of the fingers are relatively rare injuries, if left untreated, functional disabilities of the DIP joints persist. Thus, reducing the dislocation and reconstructing the articular surface at the base of the distal phalanx is crucial to allow early DIP joint motion. This study evaluates the outcomes of three patients with dorsal fracture-dislocations of the DIP joints. Two patients had a split-depression type fracture and one had an impaction type fracture, and articular involvement averaged 55.7%. They were treated with open reduction and internal fixation using a low-profile mini-plate. Dorsal instability of the DIP joint is stabilized with an extension block pin inserted into the middle phalangeal head, followed by plate fixation with the palmar approach of the distal phalanx. One patient had a concomitant dorsal fracture-dislocation of the proximal interphalangeal joint of the same finger which was treated by the same procedure. The average time from injury to surgery was 6.6 days. Postoperative outcomes were evaluated with joint motion, grip strength, Quick Disabilities of Arm, Shoulder and Hand (DASH) questionnaire score as patient-based assessment, and plain radiographs. Strickland’s scoring scale (Strickland’s score) was used to evaluate total active motion. The mean postoperative follow-up was 18.6 months. The mean postoperative DIP joint motion was 0° on extension and 71.3° on flexion, and the mean % total active joint motion was 98.3%, and Strickland's score was excellent in all cases. Grip strength was 107.7% compared to the unaffected side. The mean Quick DASH score was 3.03 points.

This study suggests that volar mini-plate fixation is an effective surgical technique for this injury, as it rigidly secures the bone fragment between the plate and the dorsal bone cortex allowing for early postoperative joint motion.

## Introduction

Dorsal fracture-dislocation of the distal interphalangeal (DIP) joint of the finger is a rare injury, but fracture morphology is akin to a dorsal fracture-dislocation of the proximal interphalangeal (PIP) joint [1]. It is usually caused by a strong axial force on the distal phalanx, and shearing forces against the middle phalangeal head result in a volar fracture of the distal phalanx base in slight flexion of the DIP joint [2]. It has also been stated that the base of the distal phalanx is not as prominent on the volar side as the dorsal side, and the cortex is thinner, making the volar fragment more prone to comminuted fractures [2].

In this injury, if left untreated, owing to the damage to the articular surface at the base of the distal phalanx, especially the palmar side, instability remains, smooth joint motion is impaired, and functional disability of the joint persists. Hence, functional recovery of the DIP joint is extremely important to allow early joint movement through the reduction of articular fracture.

It has been reported that cases with relatively small fractures of the articular surface were treated with conservative treatment using an extension block splint of the DIP joint [3]. On the other hand, in more than 40% of fractures of articular surfaces, the DIP joint is unstable, and surgical treatment is indicated [1].

Plate fixation for dorsal fracture-dislocation of the PIP joint has already been reported with satisfactory results [4,5]. The authors report on the use of this method in three patients with dorsal fracture-dislocation of the DIP joint with satisfactory results.

## Case presentation

We conducted a retrospective review of three patients with dorsal fracture-dislocation of the DIP joint who underwent surgical treatment with open reduction and internal fixation using a low-profile mini-plate with a profile height of 0.55 mm and a 1.2-mm screw diameter (Profyle Hand Standard Plating Module, Stryker, Selzach, Switzerland) through palmar approach. The patients were treated in our department between September 2020 and September 2022. Plain radiography and computed tomography (CT) were used to evaluate the fracture-dislocation of the DIP joint including the extent of articular surface involvement, depression, or impaction of the joint surface of the distal phalangeal base at the time of the initial visit.

Postoperative outcomes at the final follow-up were as follows: active range of motion (ROM) of the DIP, PIP, and metacarpophalangeal joints, grip strength, patient-based assessment using Quick Disabilities of Arm, Shoulder and Hand (DASH) questionnaire score, and plain radiographs. Recovery of ROM was assessed with total active motion (TAM) as described by the American Society for Surgery of the Hand and Strickland’s scoring scale (Strickland’s score) to assess flexor tendon repair [6]. The ratio of the TAM of the injured finger to the TAM of the healthy contralateral side is expressed as a percentage value (%TAM), and Strickland’s score is classified according to the following evaluation criteria: 85-100%, excellent; 70-84%, good; 50-69%, fair; and <50%, poor. The grip strength was measured and expressed as a percentage of the healthy contralateral side (%Grip).

Characterization of patients

**Figure 1:**
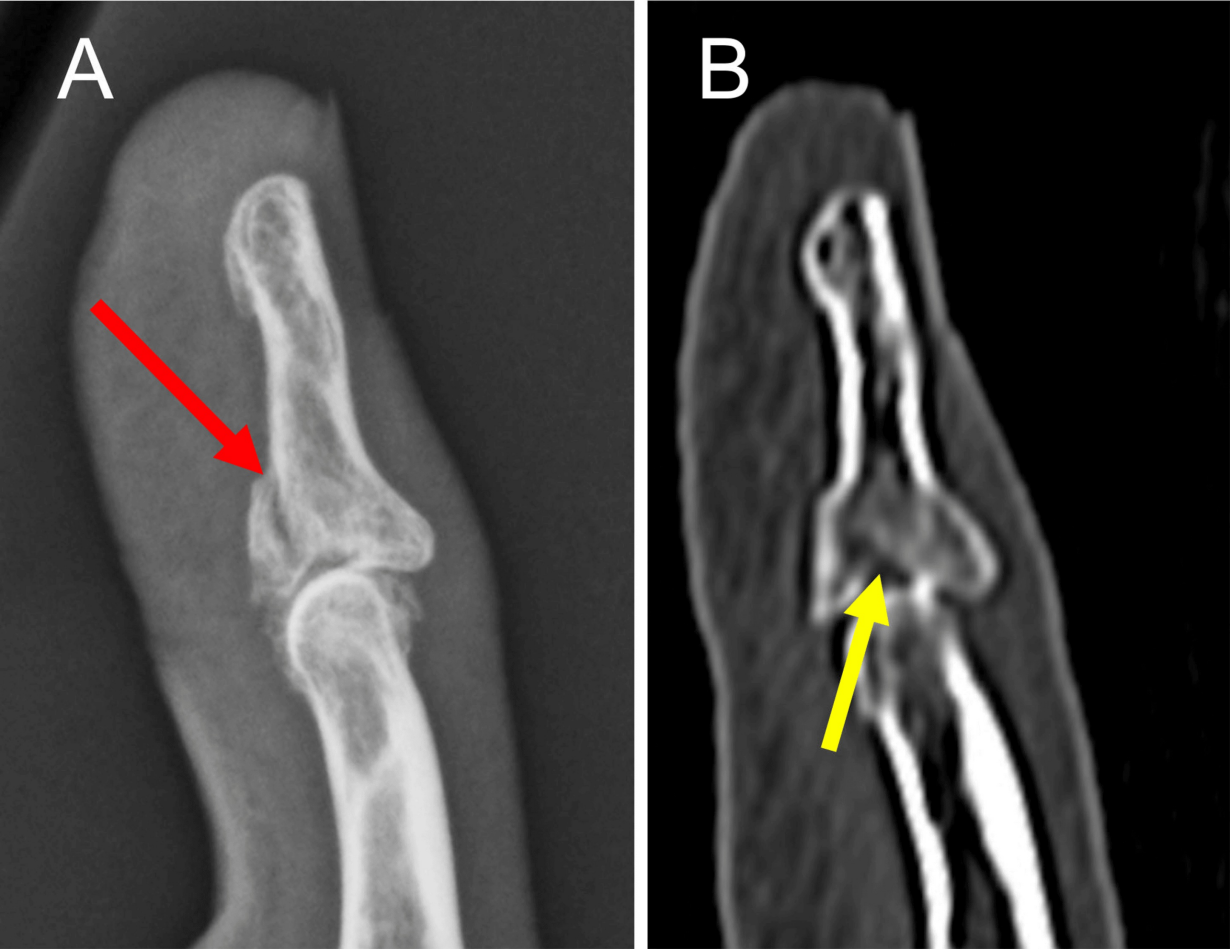
Preoperative radiograph and CT scan of Patient 1 The radiograph shows a split-depression type fracture (red arrow) with impaction of the radial articular surface in the lateral view of the right ring finger (A). The sagittal view shows an impaction of the volar fragment especially on the radial side (yellow arrow) with dorsal subluxation of the DIP joint (B). DIP: distal interphalangeal

**Figure 2:**
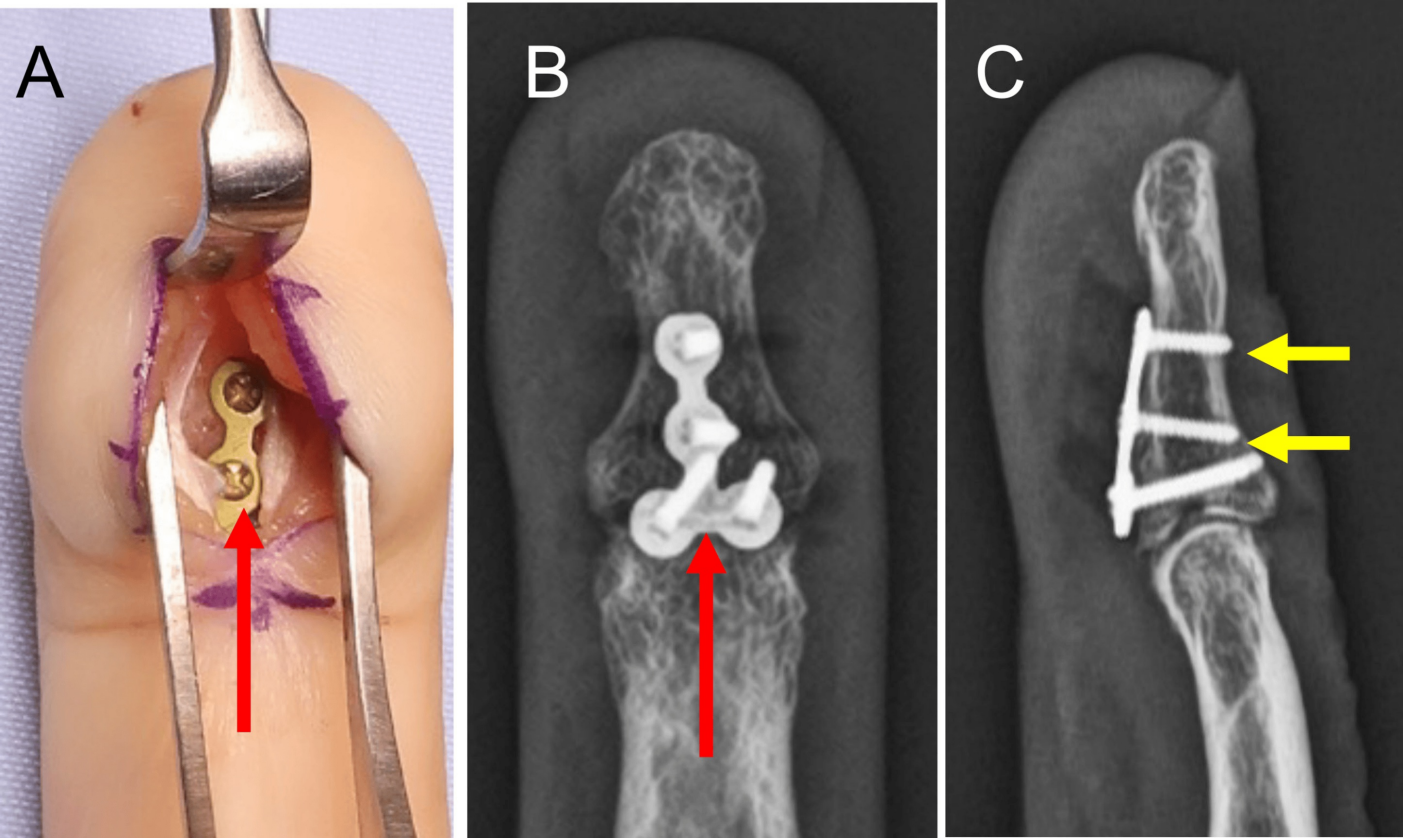
Intraoperative photograph and postoperative radiographs of Patient 1 A T-plate (red arrows) was applied at the volar surface of the distal phalanx (A). The radiographs, in both AP (B) and lateral (C) views, demonstrate the positioning of the plate and reduction of the dorsal subluxation of the DIP joint. The tip of the screws penetrates the dorsal cortex (yellow arrows), and the plate holds the bone fragment in place. DIP: distal interphalangeal

**Figure 3:**
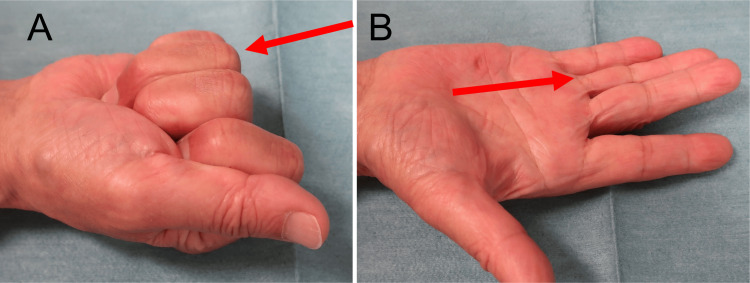
Postoperative finger motion of Patient 1 at 12 months Full grip (A) and full extension (B) of the ring finger (red arrows). The %TAM was 97.3% at 12 months postoperatively. %TAM: percentage of total active motion

Patient 1, an 83-year-old male, fell after drinking and sustained a fracture-dislocation of the DIP joint of his right ring finger. Plain radiographs and CT images showed a split-depression type volar fragment with impaction of the ulnar articular surface (Figure 1A). Articular involvement was 57.1% in the sagittal plane (Figure 1B). A four-hole T-plate fixation was performed nine days after injury (Figure 2). At 12 months postoperatively, the %TAM was 97.3% (Figure 3).

**Figure 4:**
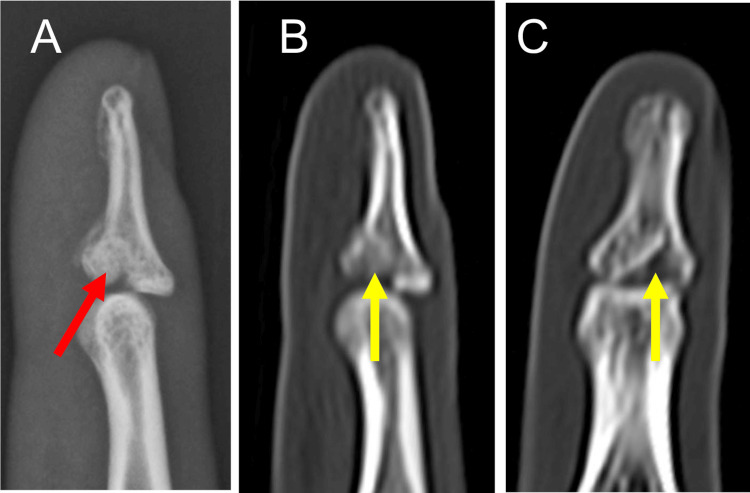
Preoperative radiograph and CT scans of Patient 2 The radiographs showing an impaction-type fracture (red arrow) in the lateral view of the right index finger (A). Sagittal view (B) and coronal view (C) show an impaction of the volar ulnar articular fragment (yellow arrows) and a dorsal subluxation of the DIP joint. DIP: distal interphalangeal

**Figure 5:**
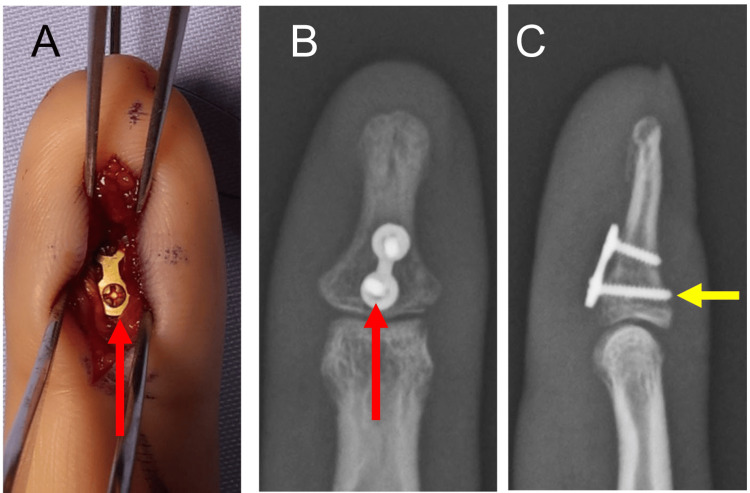
Intraoperative photograph and postoperative radiographs of Patient 2 A two-hole straight plate (red arrows) was applied at the volar surface of the distal phalanx dividing the flexor insertion (A). The radiographs, in both AP (B) and lateral (C) views, demonstrate the reduction of the dorsal subluxation of the DIP joint. The impacted articular fragment was reduced and supported by a screw (yellow arrow). DIP: distal interphalangeal

**Figure 6:**
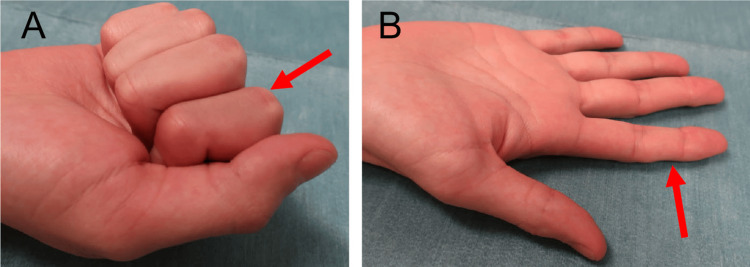
Postoperative finger motion of Patient 2 at 12 months Full grip (A) and full extension (B) of the index finger (red arrows). The %TAM was 100% at 12 months postoperatively. %TAM: percentage of total active motion

Patient 2, a 21-year-old male university student, sustained a fracture-dislocation of the DIP joint of his right index finger when he was hit by a softball pitch. Plain radiographs and CT images showed an impaction-type fracture with severe impaction of the ulnar volar side of the articular surface (Figure 4). Articular involvement was 55.2% in the sagittal plane. The operation was performed seven days after the injury. The impacted articular surface was elevated from the volar fracture site, the volar fragment was reduced, and a two-hole straight plate fixation was performed (Figure 5). The %TAM was 100% at 12 months postoperatively (Figure 6).

**Figure 7:**
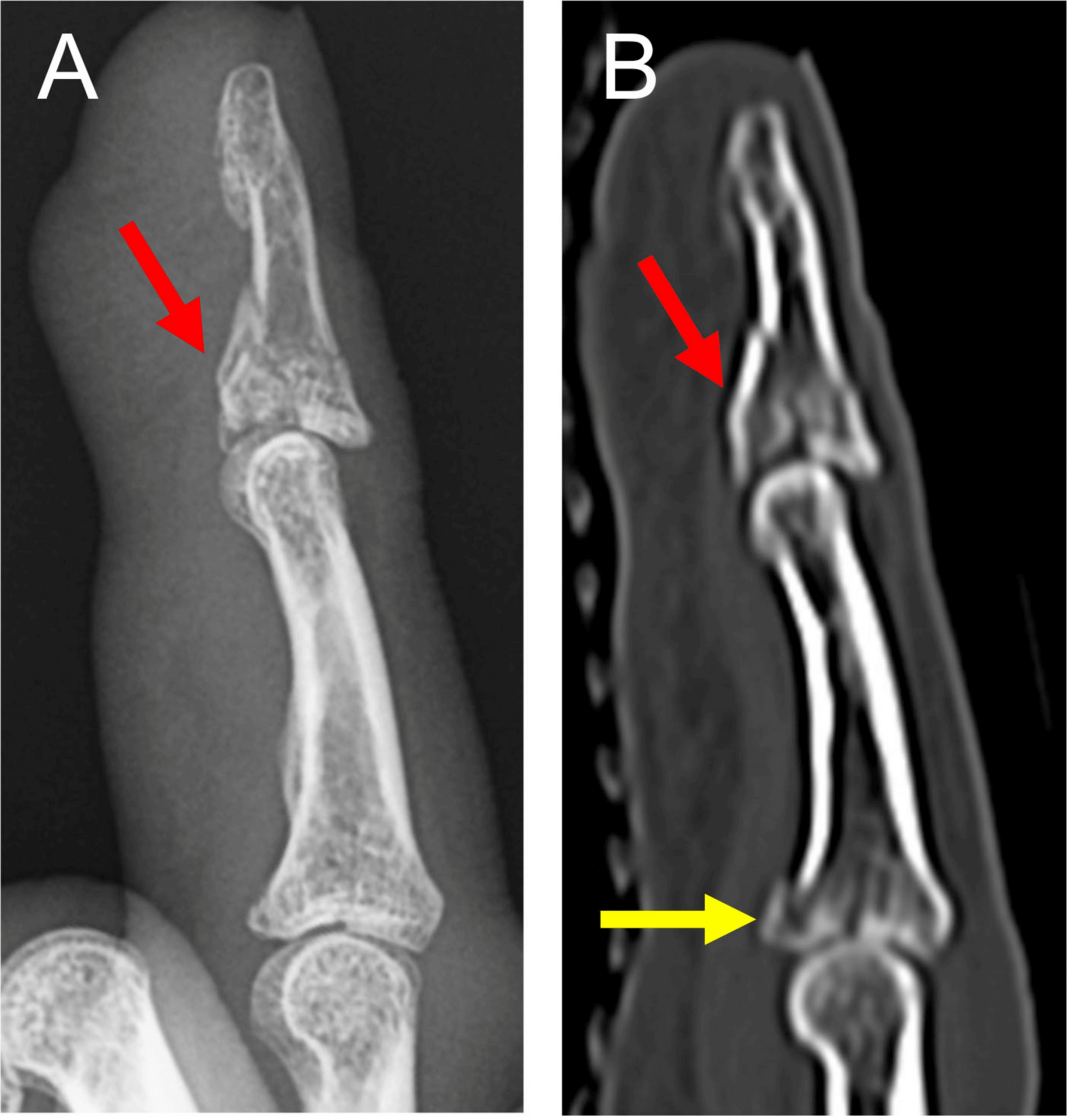
Preoperative radiograph and CT scan of Patient 3 Radiograph and CT scan showing double dorsal fracture-dislocation of the DIP and PIP joints of the right ring finger. The lateral view of the radiograph (A) and sagittal view of the CT scan (B) show a split-depression type fracture of the distal phalangeal base (red arrows). Dorsal subluxation of the PIP joint with displaced and impacted volar fragment of the middle phalangeal base (yellow arrow) is seen. DIP: distal interphalangeal; PIP: proximal interphalangeal

**Figure 8:**
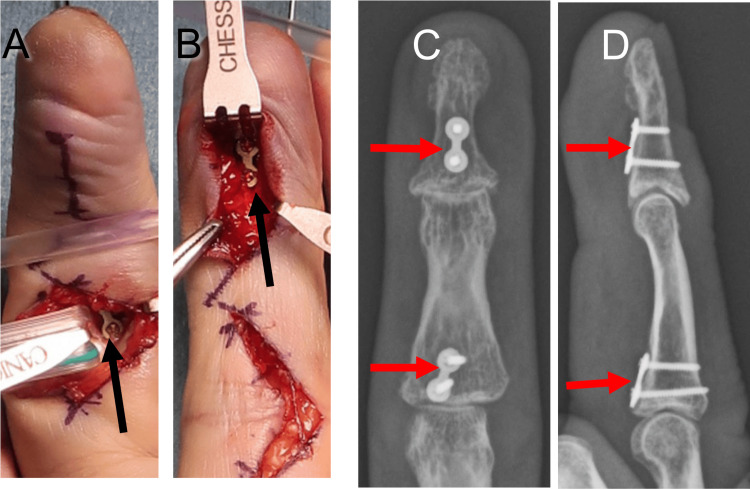
Intraoperative photograph and postoperative radiographs of Patient 3 A two-hole plate fixation (black arrows) was performed on both the distal and middle phalanges (A, B). Proximal volar cortexes of the distal and middle phalanges were fixed with plates and screws (red arrows) in buttress fashion, and dorsal subluxations were reduced (C, D).

**Figure 9:**
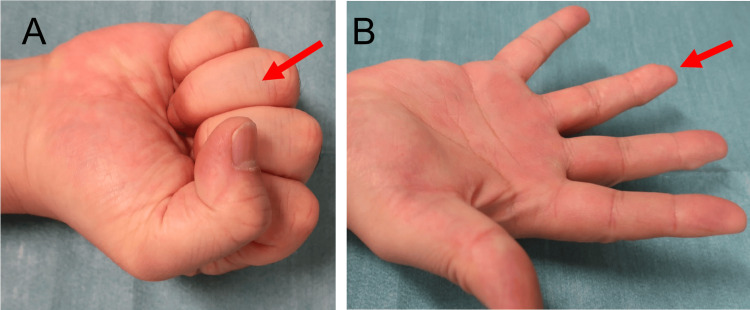
Postoperative finger motion of Patient 3 at 32 months Full grip (A) and full extension (B) of the ring finger (red arrows). The %TAM was 97.6% at 32 months postoperatively. %TAM: percentage of total active motion

Patient 3, a 52-year-old male baseball catcher, was hit by a ball and sustained a double fracture-dislocation of the DIP and PIP joints of his right ring finger (Figure 7A). In the sagittal plane, articular involvement was 54.8% at the DIP joint and 57.1% at the PIP joint (Figure 7B). The fracture of the distal phalangeal base was of split-depression type and was complicated by a dorsal fracture-dislocation of the PIP joint. Plate fixations were performed on both the distal and middle phalanges four days after injury (Figure 8). At 32 months postoperatively, the %TAM was 97.6% (Figure 9).

Surgical technique and follow-up

**Figure 10:**
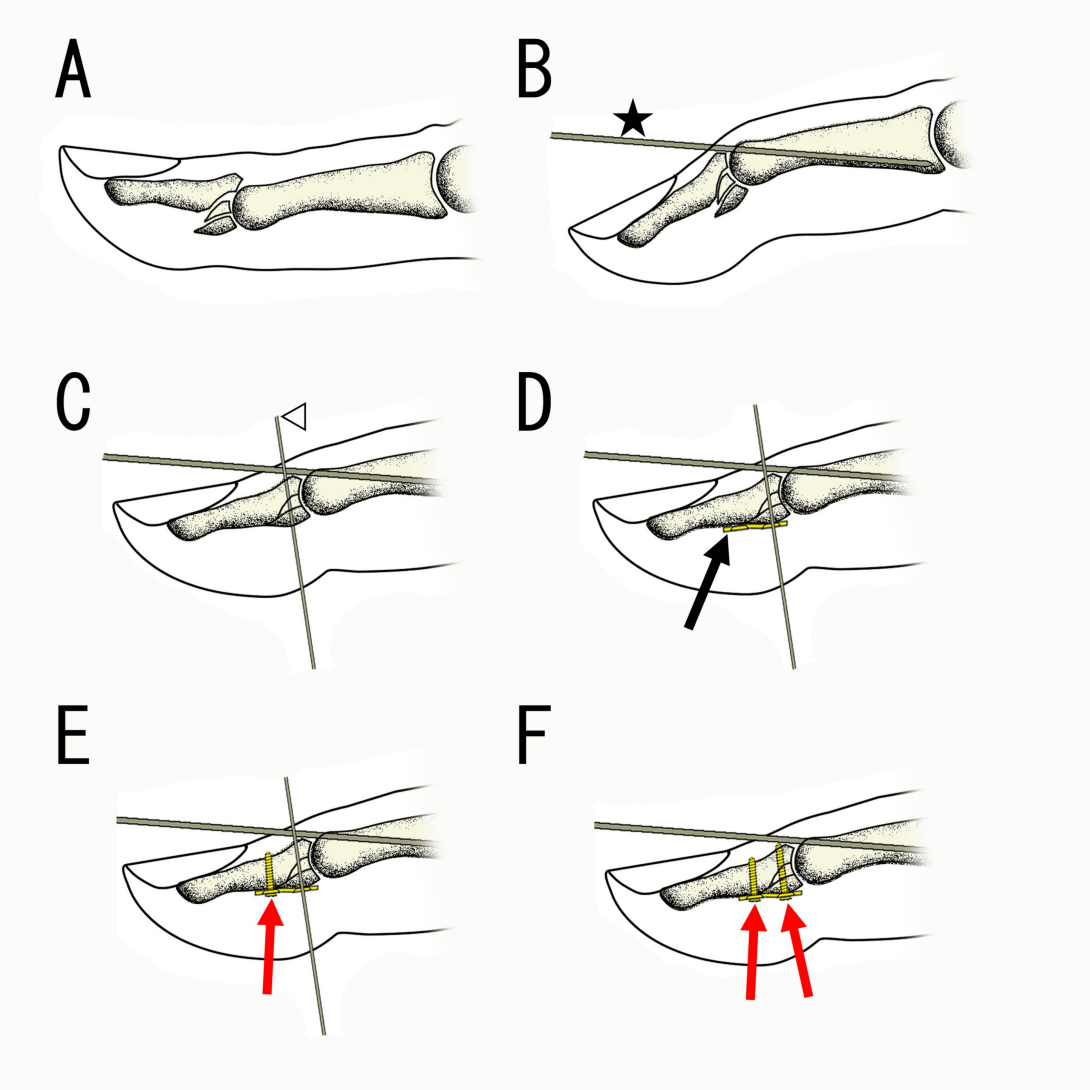
Surgical technique (A) Dorsal fracture-dislocation of the distal interphalangeal (DIP) joint. (B) Subluxation was reduced with the DIP joint flexed, and a 1.0 mm Kirschner wire (K-wire) (★) was inserted into the dorsal edge of the middle phalangeal head. (C) The DIP joint was extended, and the volar fragment was reduced and temporarily fixed with a 0.7 mm K-wire (◁). (D) A plate (black arrow) was applied over the fragment passing the wire through the proximal hole. (E) A screw (red arrow) was inserted in the distal hole to exert a buttress effect. (F) Then, the temporarily fixed K-wires were replaced with a screw (red arrow). Figures were created by Masayoshi Ikeda.

The operation was performed on Patients 1 and 2, who underwent digital nerve block anesthesia with a finger tourniquet, and Patient 3 underwent general anesthesia with an upper arm pneumatic tourniquet by the same highly experienced hand surgeon. A high-resolution mini-image intensifier (Fluoroscan InSight-FD mini C-arm system, Hologic, MA, USA) was used during the procedure. First, the DIP joint was flexed to reduce dorsal subluxation, and a 1.0 mm Kirschner wire (K-wire) was percutaneously inserted into the dorsal edge of the middle phalangeal head as an extension block (Figures 10A-10B). Next, the DIP joint was extended, and the palmar aspect of the distal phalanx was explored via a short midline palmar incision. The flexor digitorum profundus (FDP) tendon attachment was divided in the midline and the palmar aspect of the base of the distal phalanx was explored. Then, the volar fragment was reduced and temporally fixed with 0.7 mm K-wires (Figure 10C). When depressed articular fragments are present, they should be reduced through the volar fracture site. The implant was a nonlocking plate with a profile height of 0.55 mm and a screw diameter of 1.2 mm for internal fixation. The plate was cut down sufficient to cover both the intact cortex and the fracture fragment, and placed as a buttress, passing the wires through the holes of the plate (Figure 10D). After screwing the distal hole in the plate (Figure 10E), the temporarily fixed K-wires were replaced with a screw (Figure 10F). The extension block was removed and the wound was closed with 5-0 nylon sutures. An aluminum splint was applied from the tip to the proximal phalanx in extension. The active motion was initiated after one week of immobilization.

Results

Three patients with dorsal fracture-dislocation of the DIP joint were treated using this technique, and they were all male with a median age of 56 years. One (Patient 2) had a dorsal fracture-dislocation of the DIP joint of the index finger and two (Patients 1 and 3) had of the ring fingers. An average of 55.7% of articular involvement of the DIP joint was fractured. Patient 3 had a concomitant dorsal fracture-dislocation of the PIP joint of the same finger. In Patient 1, fixation was achieved using a four-hole T-plate. In Patients 2 and 3, fixation was achieved with a two-hole straight plate. In Patient 3, the fracture of the middle phalangeal base was also fixed with a two-hole straight plate. There were no intraoperative complications. Patient demographic data are presented in Table 1.

**Table 1:** Patient demographic data Delay: time from injury to surgery Patient 3 had dorsal fracture-dislocation of the PIPJ, which was reduced and fixed with a two-hole straight plate. DIP: distal interphalangeal; PIPJ: proximal interphalangeal joint

Patient no.	Age (years)	Sex	Laterality	Finger	Delay (days)	Fracture type	Articular involvement of DIP joint (%)	Implant
1	83	Male	Right	Ring	9	Split-depression	57.1	Four-hole T-plate
2	21	Male	Right	Index	7	Impaction	55.2	Two-hole straight plate
3	56	Male	Right	Ring	4	Split-depression	54.8	Two-hole straight plate

The mean postoperative follow-up period was 18.6 (12-32) months. All patients achieved bone union, and finger function was restored satisfactorily. Patient follow-up period and their results are presented in Table 2.

**Table 2:** Patient follow-up period and outcomes Q-DASH; Quick Disabilities of Arm, Shoulder and Hand (DASH) questionnaire score; DIPJ: distal interphalangeal joint; PIPJ: proximal interphalangeal joint; MPJ: metacarpophalangeal joint; %TAM: percentage of total active motion %TAM=((total active range of motion (DIPJ+PIPJ+MPJ) of affected side)/(total active range of motion (DIPJ+PIPJ+MPJ) of contralateral side))×100 %Grip=((grip power of affected side)/(grip power of contralateral side)) ×100

Patient no.	Follow-up period (months)	Active range of motion (ﾟ)			%TAM (%)	%Grip (%)	Strickland’s score	Q-DASH (point)
		DIPJ	PIPJ	MPJ				
1	12	0~63	0~103	0~96	97.3	96	Excellent	9.1
2	12	0~83	0~106	0~90	100	125	Excellent	0
3	32	0~63	0~101	0~81	97.6	102	Excellent	0

## Discussion

It is necessary to rigidly fix the fracture of the volar aspect of the base of the distal phalanx and restore the joint surface to its anatomical configuration in the management of the dorsal or intraarticular fracture-dislocation of the DIP joint. Thus, it is vital to allow the full range of flexion and extension of the DIP joint in the earlier period immediately after the operation to prevent joint contracture. This type of finger trauma is much rarer than bony mallet injury with a dorsal fracture fragment and reports elucidating this type of trauma remain limited [1,2].

Various conventional treatment methods have been reported. One method is to hold the DIP joint in flexion with an extension block splint or extension block pin to reduce the dorsal subluxation and wait for soft tissue healing including palmar fractures. Hamer and Quinton reported that conservative treatment with an extension block splint led to a 73% ROM of the DIP joint with a 5° flexion contracture and an average residual dorsal shift of 1.8 (1.5-3) mm after and an average of 4.3 weeks of splint immobilization [3]. However, conservative treatment such as extension block splint is rendered difficult when the joint surfaces are comminuted. There is a case report of a patient treated with an extension block pin after fracture reduction with the lateral approach, which required five weeks of immobilization with pin insertion and did not allow a full ROM immediately after surgery [7].

Horiuchi et al. [2] performed a temporal immobilization with a K-wire or tension band wiring in cases with large bone fragments, bone grafting, or other bone substitutes in comminuted fracture cases, with a four-week DIP joint immobilization. Eight of 12 patients had a dorsal fracture-dislocation of the DIP joint except for the thumb, and one patient had a primary joint fusion 46 days after the injury. Among the remaining seven patients, three reported poor joint ROM. In the article, the authors stated that the proximal volar ridge of the distal phalanx is less prominent and has a thinner cortex than the dorsal side, which is the reason why the volar fragment is more prone to comminuted than a standard bony mallet. Furthermore, because the tendon is not attached to the volar fragment, as in the case of dorsal fracture-dislocation of the PIP joint, palmar stability of the DIP joint can be achieved when there is a union of the volar fragment and soft tissue healing. However, the anatomical congruity of the joint surface is considered crucial for a smooth full-range sliding motion of the joint. Kobayashi and Fukusawa [8] reported two cases treated with an external fixation using a K-wire for this injury. The ROM of the DIP joint in each case was 62.5% and 76% relative to the unaffected side. The authors reported that early joint motion with an external fixator promotes damaged soft tissue remodeling and joint stability.

Nakago et al. [9] reported 16 cases of simultaneous fracture-dislocation of the DIP and PIP joints. Among them, there were four cases of double hyperextension injury such as case 3 in this series, and one of them had poor results. The authors concluded that PIP joint injuries are often preferentially treated over DIP joint injuries and that inadequate stability of the PIP joint may delay DIP joint mobilization. Rettig et al. [10] reported 10 cases (four thumb IP joints and six finger DIP joints) who underwent volar plate advancement arthroplasty and K-wire fixation for chronic dorsal fracture-subluxation. All cases had a flexion contracture averaging 12 degrees, although had stable joint motion.

The surgical technique used in this series can be applied via a midline incision on the palmar aspect of the distal phalanx. The FDP tendon attachment on the volar aspect of the distal phalanx is striated so that the mini-plate can be applied, and the tendon should not be stripped off. This technique is easier than internal fixation of the dorsal fracture-dislocation of the PIP joint as avoiding the tendon sheath or flexor tendon in the case of dorsal fracture-dislocation of the DIP joint is not required. The volar fragment can be firmly fixed with a plate, allowing full ROM early in the postoperative period. One of the limitations of this study is the limited number of cases; therefore, the fracture type classification for which the method is indicated is not discussed.

## Conclusions

We employed volar plate fixation for dorsal fracture-dislocation of the DIP joint as a surgical treatment for dorsal fracture-dislocation of the PIP joint. This method provides good fixation by supporting the volar fragment at the base of the proximal phalanx between the plate and the dorsal cortex and allows joint movement in the early postoperative period.
